# Enhanced Spatial Mapping of Mouse Gastric Muscle Layers Using a Modified Swiss Roll Technique

**DOI:** 10.3791/69673

**Published:** 2025-11-25

**Authors:** Nick M. Huynh, Srinidhi R. Babu, Egan L Choi, Tamas Ordog, Yujiro Hayashi

**Affiliations:** 1 Enteric Neuroscience Program and Department of Physiology and Biomedical Engineering, Mayo Clinic College of Medicine and Science; 2 Gastroenterology Research Unit, Division of Gastroenterology and Hepatology, Department of Medicine, Mayo Clinic College of Medicine and Science; 3 Division of Gastroenterology and Hepatology, Department of Medicine, Mayo Clinic College of Medicine and Science

## Abstract

Spatial “omics” technologies provide unprecedented opportunities to study the biology and interactions of individual cells of the gastric neuromuscular apparatus in their native tissue environment. However, the successful application of these approaches and the assessment of structural and molecular differences across the fundus, corpus, and antrum require consistent sampling and tissue orientation. Here, we report a modified “Swiss roll” technique tailored to the mouse stomach, which allows all major anatomical regions to be embedded and visualized in a single cryosection. This approach emphasizes flat fixation and careful positioning to generate sections with reproducible orientation and quality. This study also optimized immunofluorescence staining conditions to ensure high-quality visualization of cellular markers. This protocol enables comprehensive, side-by-side comparisons of gastric regions while minimizing tissue loss and experimental variability. By enhancing tissue preservation and spatial orientation, this method offers a practical and scalable tool for researchers studying gastric biology in both physiological and pathological contexts.

## Introduction

The stomach is a complex, J-shaped organ that connects the esophagus to the duodenum^1^. It is divided into three main anatomical regions: the fundus, corpus, and antrum^1^. Each region of the gastric tunica muscularis plays a distinct physiological role, contributing to key gastric functions such as accommodation, compliance, food trituration, and emptying^1,2^. Studying changes in gastric physiology and pathology requires detailed analysis of these regions and their cellular architecture.

To visualize large areas of tissue, researchers often adapt the “Swiss roll” technique, originally developed for the murine intestine^3,4,5,6^. This study modified the Swiss roll technique for use with the murine stomach to establish a new protocol. The stomach is first separated into its anatomical regions and fixed in a flat orientation. The tissue is then carefully rolled and processed for cryosectioning. Improper Swiss rolling can damage fragile epithelial structures, particularly in the intestine, leading to poor tissue quality for immunostaining. Preserving well-fixed, properly oriented tissue with intact cellular structures is essential for high-resolution imaging. This work presents a new method for generating Swiss rolls that include all gastric regions within a single cryoblock. Additionally, an optimized immunofluorescence staining protocol is described for the gastric tunica muscularis, enabling detailed evaluation of gastric function. This protocol provides a comprehensive guide for obtaining high-quality immunofluorescence images through careful preparation of gastric tissue, Swiss roll processing, and staining. By preserving the intricate morphology of the gastric tunica muscularis, this method facilitates a deeper understanding of its structure and function in both health and disease.

## Protocol

Animal experiments were conducted with the guidelines outlined in the National Institutes of Health Guide for the Care and Use of Laboratory Animals. The protocols were approved by the Mayo Clinic Institutional Animal Care and Use Committee (A48315–15). Mayo Clinic’s animal care and use programs and facilities have been reviewed and fully accredited by the Association for Assessment and Accreditation of Laboratory Animal Care International (AAALAC). The reagents and the equipment used are listed in the Table of Materials.

### 1. Swiss roll technique for paraformaldehyde (PFA)-fixed gastric cryosections

**Figure 1:**
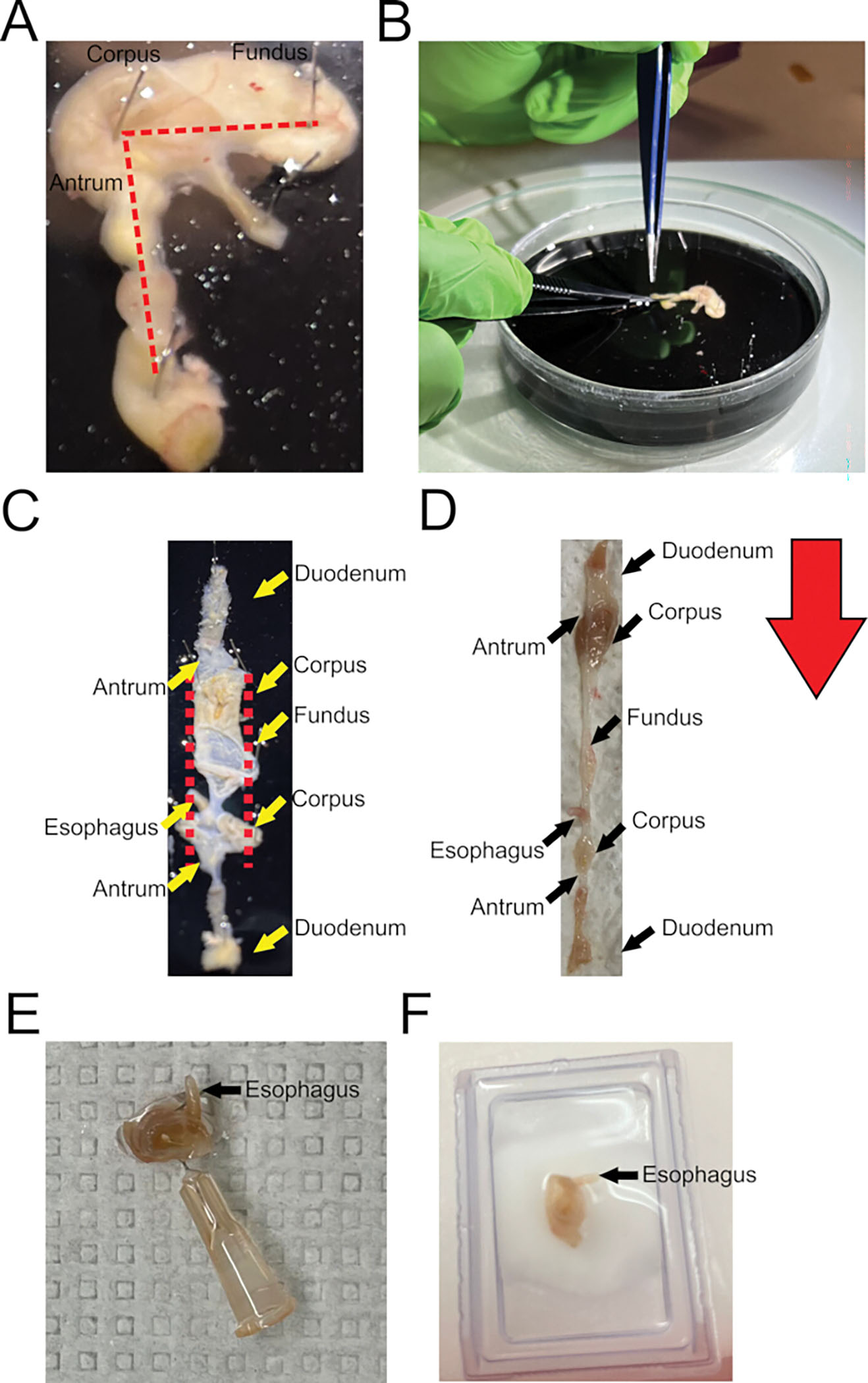
Preparation of “Swiss roll” of the mouse stomach for cryosectioning. (**A**) The whole stomach is placed in a black silicone-coated dissection dish filled with cold phosphate-buffered saline (PBS) immediately after removal to preserve tissue integrity. (**B**) The stomach is opened along the midpoint of the duodenum. (**C**) The stomach is gently stretched and pinned in the dish to lay flat without applying excessive tension. A red dashed line is outlined along the tissue to indicate where it should be trimmed to facilitate rolling. (**D**) The opened and fixed stomach is placed on PBS-moistened filter paper in preparation for rolling into a compact configuration. The red arrow shows the direction of stomach rolling. (**E**) Tissue pieces are embedded in optimal cutting temperature (O.C.T.) compound and frozen on dry ice in preparation for cryosectioning. (**F**) The frozen blocks are stored at −80 °C and cryosectioned at 5-μm thickness for downstream analysis. Please click here to view a larger version of this figure.

1. Anesthetize a C57BL/6J mouse (8–10 weeks old) *via* carbon dioxide (CO_2_) inhalation for at least 3 min, followed by cervical dislocation to ensure euthanasia (following institutionally approved protocols).
2. Cut the esophagus, duodenum, and the ventral and dorsal mesogastria to remove the stomach.
3. Place the stomach in a silicone-coated dish containing cold phosphate-buffered saline (PBS). Pin the esophagus, fundus, corpus, and duodenum to serve as orientation references (Figure 1A). The cutting direction is indicated by a red dashed line in Figure 1A.
4. Carefully remove any attached fat using dowel scissors and forceps.
5. Cut along the ventral mesentery of the duodenum and the stomach up to the pin at the corpus, then gently flatten the tissue (Figure 1B).
6. Use a transfer pipette filled with PBS to flush out any residual food from the gastric lumen.
7. Gently stretch the opened stomach to achieve a flat orientation (Figure 1C).
  **CAUTION:** Avoid overstretching (i.e., more than 110% of the original dimensions) to preserve tissue architecture.
8. Immerse the stomach in cold 4% PFA for 2 min to lightly fix the tissue and facilitate rolling.
9. Pin the tissue flat again after the brief fixation.
10. Trim the stomach along the dashed outline to create a uniform shape suitable for rolling (Figure 1C).
11. Place the trimmed gastric tissue on PBS-moistened filter paper (Figure 1D).
12. Roll the tissue into a Swiss roll configuration and secure it by inserting a toothpick through the center.
  **CAUTION:** Ensure the mucosal side is facing upward during the rolling process. Hold the duodenal end with forceps in one hand while guiding the toothpick with the other, following minor modifications of published methods.
13. Once rolled, insert a 30-G needle to secure its shape and bend it at a 90° angle to stabilize the rolled stomach (Figure 1E).
14. Fix the rolled tissue in 4% PFA at 4 °C for 6 h.
15. Transfer the fixed tissue to 30% sucrose in PBS and incubate at 4 °C for 12–16 h for cryoprotection and prevention of ice crystal formation.
16. Gently remove the stabilizing needle.
17. Embed the tissue in optimal cutting temperature (O.C.T.) compound using disposable base molds, then freeze on dry ice (Figure 1F).
18. Once fully frozen, store the mold at −80 °C.
  **CAUTION:** Avoid air bubbles in the O.C.T., especially around the tissue, as they interfere with sectioning. Tissue blocks can be stored at −80 °C for at least one year.
19. Before sectioning, equilibrate the block at −20 °C for at least 1 h.
20. Section the tissue at 5-μm thickness using a cryostat.
  **CAUTION:** A thickness of 5–12 μm is generally suitable for murine gastric sections^7,8^, and can be adjusted based on experimental needs. Sections can be stored at −20 °C for up to 3 months.

### 2. Immunofluorescent staining of gastric muscle tissue

1. Using a hydrophobic barrier pen, draw a water-repellent barrier around each tissue section on the microscope slide.
2. Wash the sections with cold PBS for 5 min to dissolve residual O.C.T. compound.
3. Block non-specific binding by incubating the sections with 1% bovine serum albumin (BSA) in PBS for 1 h at room temperature (RT; 21°–23 °C).
4. Remove the 1% BSA using suction, then wash the sections three times with cold PBS for 10 min each at RT (21–23 °C).
5. Further block non-specific binding by incubating the sections with a mouse-on-mouse IgG blocking solution in PBS for 1 h at RT (21–23 °C), which reduces the binding of endogenous IgG and improves specificity when using mouse antibody IgG.
6. Remove the blocking solution using suction, then wash the sections three times with cold PBS for 10 min each at RT (21–23 °C).
7. Repeat BSA blocking for 1 h at RT (21–23 °C).
8. Incubate the sections with primary antibodies. Anoctamin-1 (ANO1), KIT, beta-tubulin class III (TUBB3) diluted in cold 1% BSA with 0.3% Triton X-100. In a separate experiment, use myosin heavy chain 11 (MYH11), E-cadherin, and KIT. Incubate overnight (12–16 h) at 4 °C.
9. Remove the primary antibody solution using gentle suction. Wash the sections with cold PBS three times for 10 min each at RT (21–23 °C).
10. Incubate the sections with secondary antibodies. Alexa Fluor (AF) 488 anti-rabbit IgG, AF555 anti-mouse IgG, and AF647 anti-goat IgG (all at 5 μg/mL; 1:400) in 1% BSA for 1 h at RT (21–23 °C).
11. Remove the secondary antibody solution using suction, then wash the sections three times with cold PBS for 10 min each at RT (21–23 °C).
12. Stain the sections with 4′,6-diamidino-2-phenylindole (DAPI, at 1:300 dilution) for 1 h at RT (21°−23 °C).
  **CAUTION:** Neuronal nuclei typically show weaker DAPI signals than other GI cell types; a minimum 1 h incubation is recommended for optimal visualization.
13. Rinse the sections once with ultrapure water to remove residual salts.
14. Mount the slides using Antifade Mountant.
15. Seal the corners of the coverslip with non-fluorescent lacquer nail polish. Remove any excess mounting medium using gentle suction. Then, seal the entire perimeter of the coverslip.

### 3. Immunofluorescence imaging using a confocal microscope

**Figure 2:**
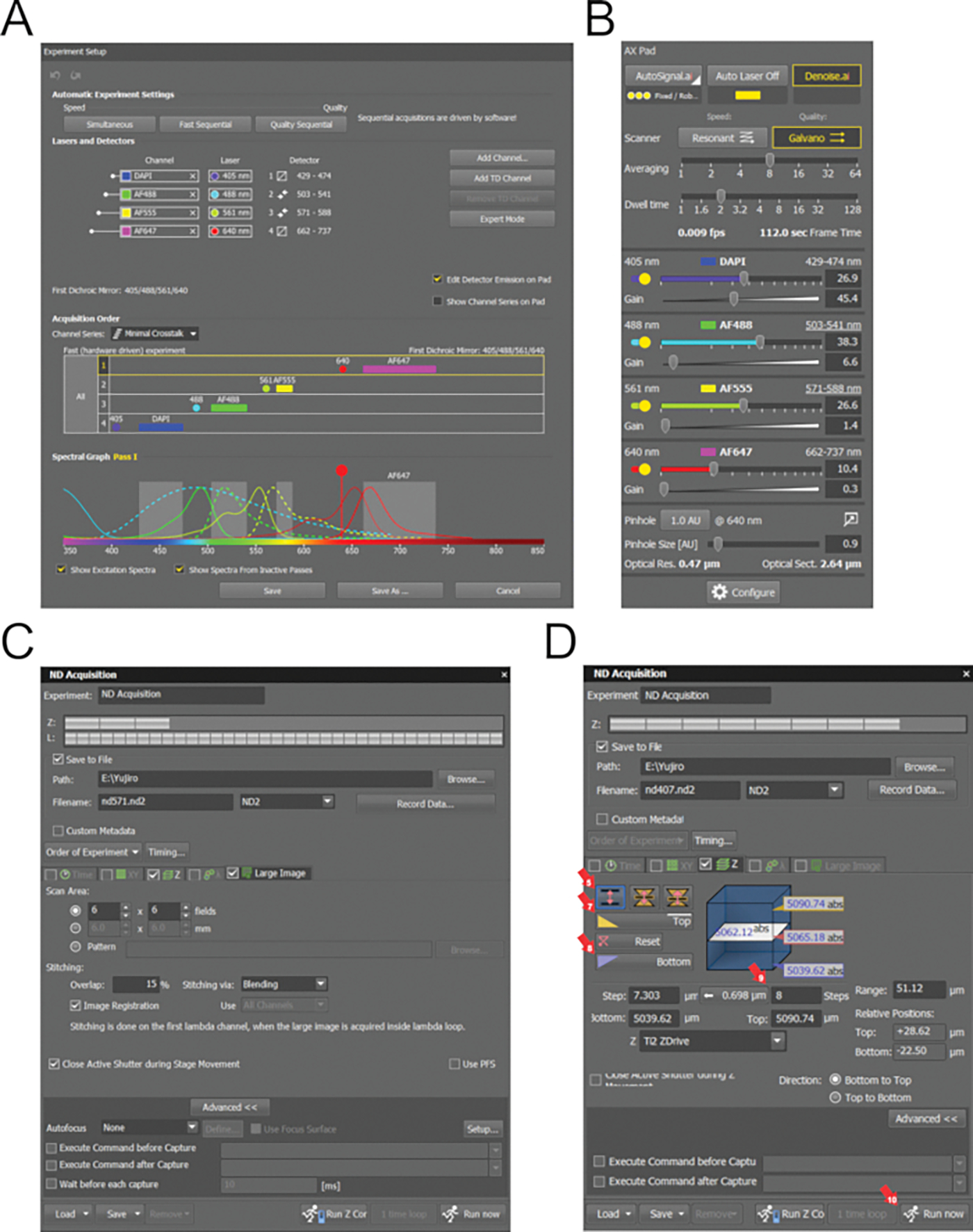
ND Acquisition setup and saturation visualization during Z-stack imaging. (**A**) Laser and detector settings configured in the confocal imaging software for multi-channel spectral imaging. Four fluorophores are used: 4′,6-diamidino-2-phenylindole (DAPI), Alexa Fluor 488 (AF488), Alexa Fluor 555 (AF555), and Alexa Fluor 647 (AF647), each paired with corresponding excitation lasers and emission detectors. The spectral graph displays excitation and emission curves, allowing adjustment to minimize crosstalk and optimize signal detection. (**B**) Exposure settings are adjusted individually for each fluorophore to avoid pixel saturation and preserve detail in high-intensity regions. (**C**) Large image acquisition setting (e.g., 6 × 6 fields) enables visualization of the entire gastric Swiss roll; image field size should be adjusted according to the size and compactness of the rolled tissue. (**D**) ND Acquisition window showing Z-stack parameter settings. The Z range is defined by selecting the top and bottom positions of the gastric muscle, with eight steps configured to capture the full depth of the tissue for three-dimensional (3D) analysis. Please click here to view a larger version of this figure.

1. Set up multi-channel Z-stack acquisition in the confocal imaging software by configuring lasers and detectors for DAPI, AF488, AF555, and AF647, ensuring spectral separation and avoiding signal saturation.
  **CAUTION:** Adjust laser power and exposure time individually for each fluorophore to prevent crosstalk and maintain signal within the dynamic range across all channels (Figure 2A).
2. Set exposure parameters in the confocal imaging software to avoid pixel saturation and preserve signal detail in high-intensity regions (Figure 2B).
  **CAUTION:** Ensure that signals in all channels remain within the dynamic range and do not reach saturation.
3. To capture the entire gastric Swiss roll, use a large image acquisition setting (e.g., 6 × 6 fields) (Figure 2C); however, this setting should be adjusted depending on the overall size and tightness of the rolled tissue.
4. Acquire 3D images using Z-series acquisition in the confocal imaging software. The detailed workflow for N-dimensional (ND) acquisition is illustrated in Figure 2D.
5. Select the **Z-stack** option to enable Z-series imaging.
6. Define the Z-range by setting the top and bottom focal positions encompassing the full thickness of the whole stomach.
7. Bring the top of the stomach into focus and click on the **Top** button to set the upper Z-limit.
8. Adjust the focus to the bottom of the gastric muscle and click on the **Bottom** button to set the lower Z-limit.
9. Set the number of Z-stack steps to 8 to span the full depth of the gastric muscle.
10. Click on **Run now** to initiate Z-stack image acquisition.
11. Generate maximum-intensity projections for individual and merged fluorescence channels to visualize three-dimensional data in a two-dimensional image.

## Representative Results

**Figure 3:**
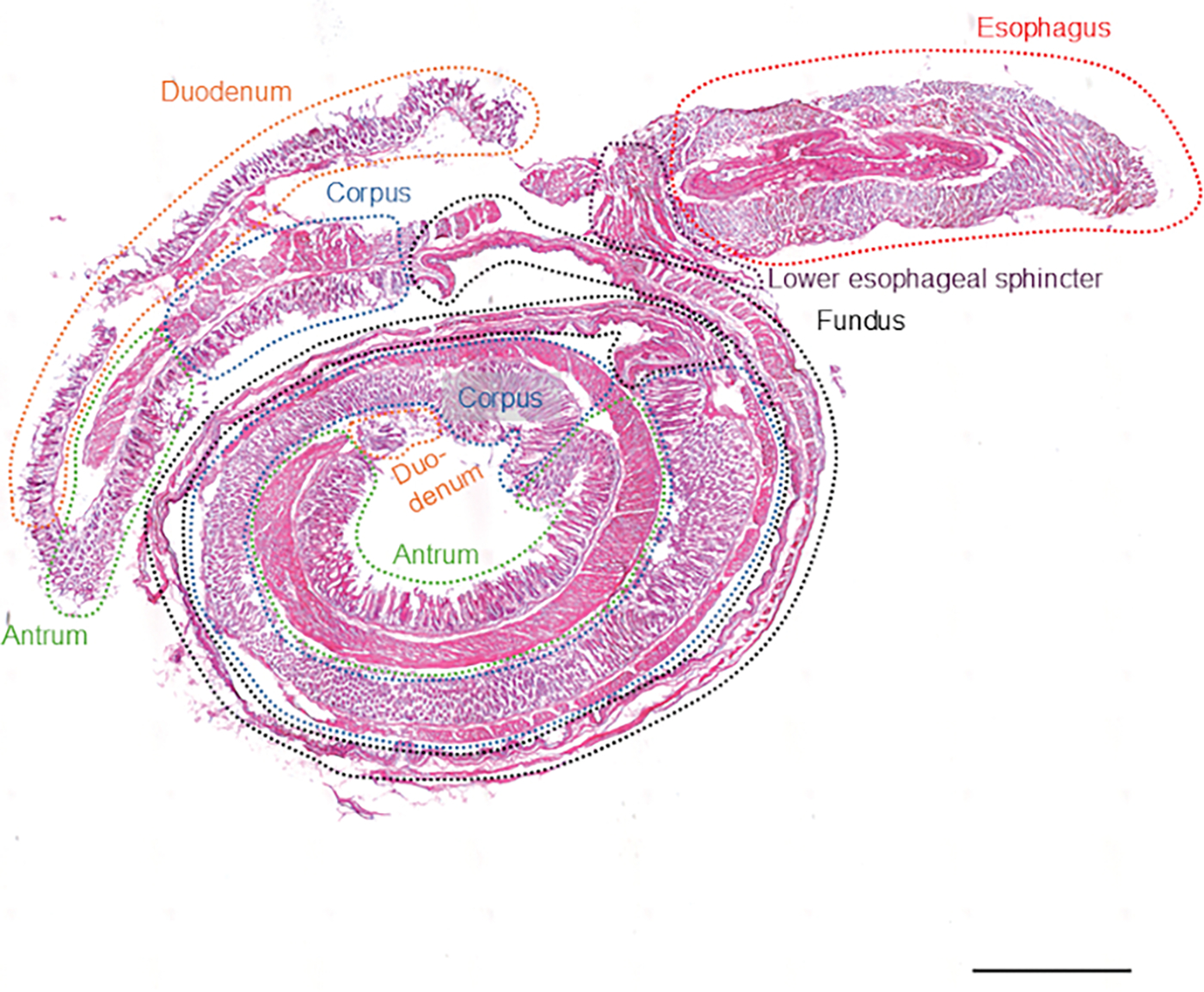
Image of a hematoxylin- and eosin-stained mouse stomach Swiss roll. Tile scanning demonstrates the ability to visualize the entire gastric Swiss roll from an 8-week-old female C57BL/6J mouse. Major anatomical regions are indicated, including the esophagus (red dotted line), lower esophageal sphincter (purple dotted line), fundus (black dotted line), corpus (blue dotted line), antrum (green dotted line), and duodenum (orange dotted line). Scale bar: 1000 μm. Please click here to view a larger version of this figure.

**Figure 4:**
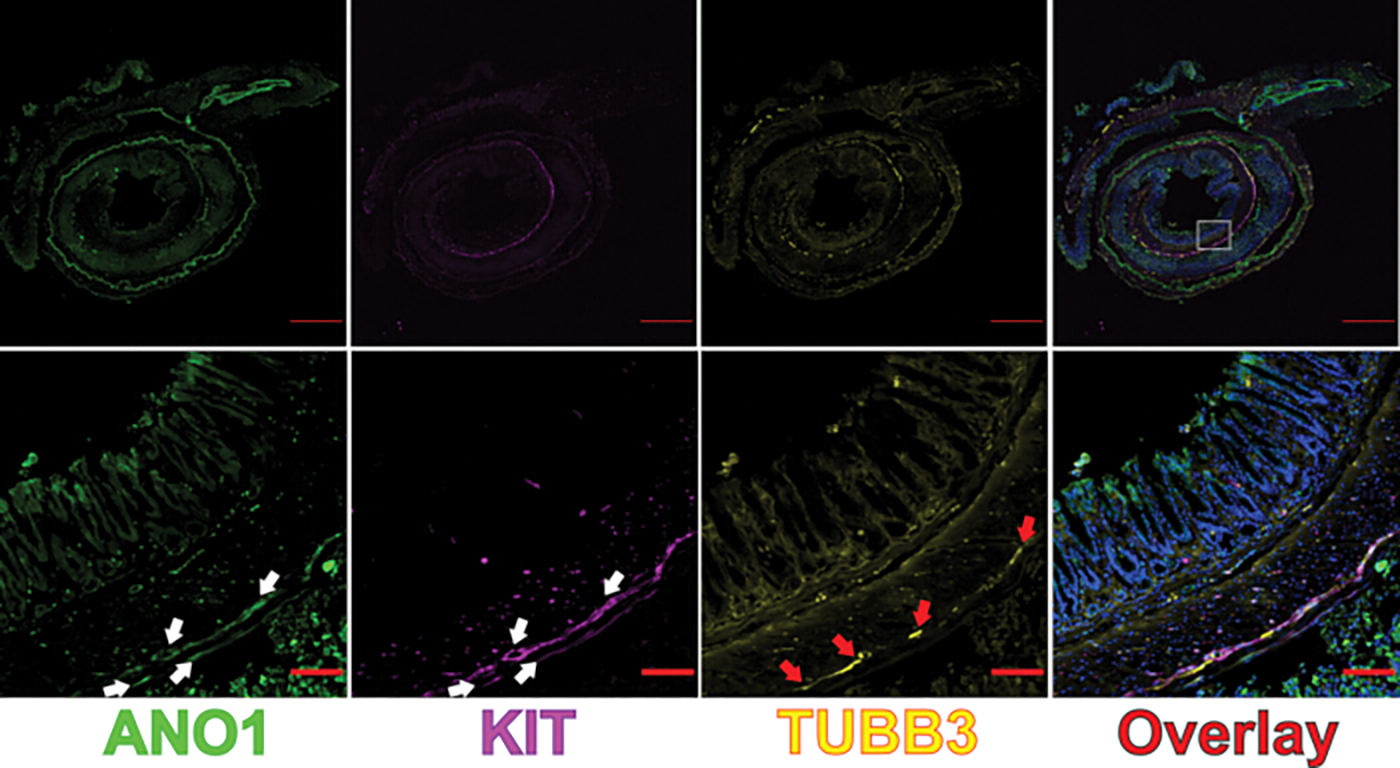
Confocal images of a Swiss roll of a whole murine stomach: ANO1, KIT, TUBB3. *Top panels*: Representative stitched confocal images of a gastric Swiss roll from an 8-week-old female C57BL/6J mouse, showing immunostaining for ANO1 (green), KIT (cyan), TUBB3 (yellow), and nuclei (blue). The entire rolled stomach, including fundus, corpus, and antrum, is visualized. Scale bars: 1000 μm. *Bottom panels*: Magnified views of the region outlined by the white dashed box in the top panels, highlighting cellular details of interstitial cells of Cajal (ANO1^+^ and KIT^+^; yellow arrows) and enteric neurons (TUBB3^+^; red arrows). Scale bars: 100 μm. Please click here to view a larger version of this figure.

**Figure 5:**
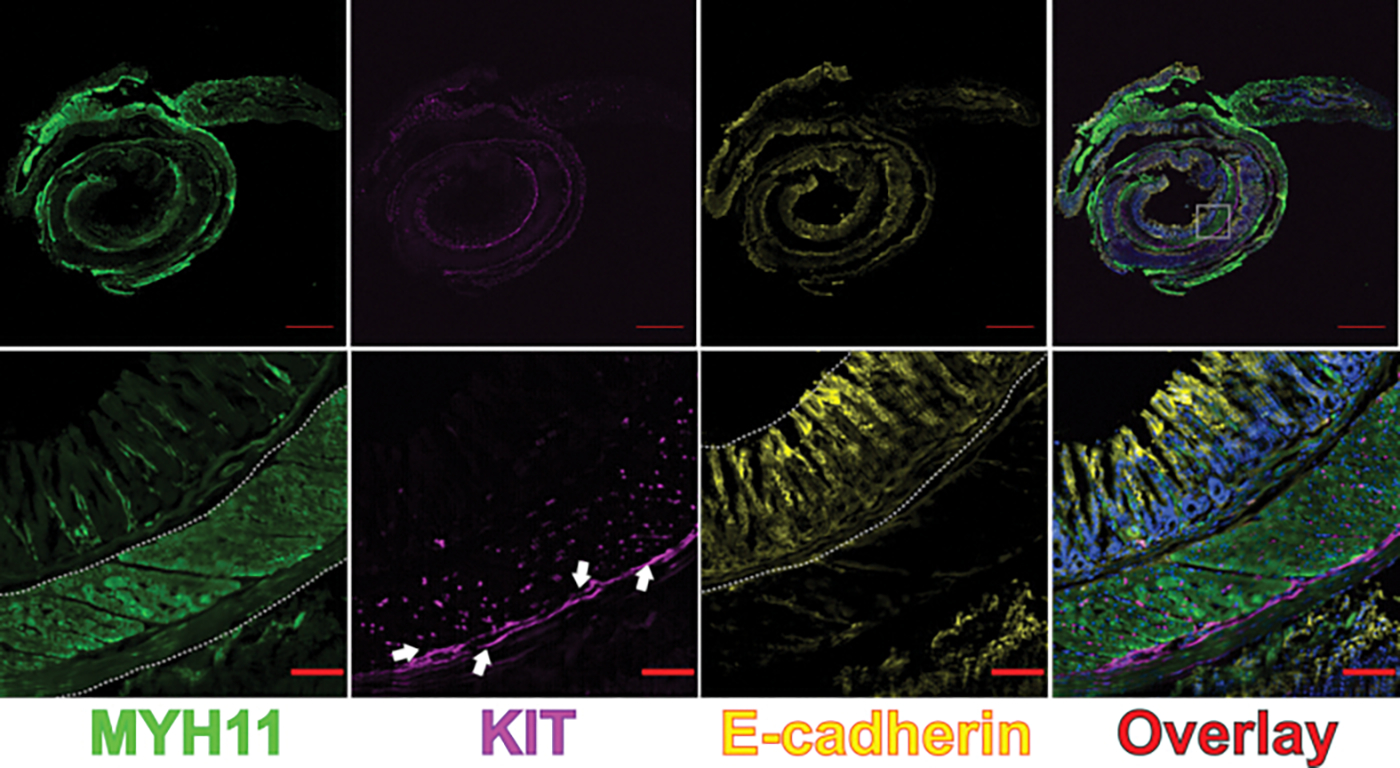
Confocal images of a Swiss roll of a whole murine stomach: MYH11, KIT, E-cadherin. *Top panels*: Representative stitched confocal images of a gastric Swiss roll from an 8-week-old female C57BL/6J mouse, showing immunostaining for MYH11 (green), KIT (cyan), E-cadherin (yellow), and nuclei (blue). The entire rolled stomach, including fundus, corpus, and antrum, is visualized. Scale bars: 1000 μm. *Bottom panels*: Magnified views of the region outlined by the white dashed box in the top panels. White dotted lines delineate the mucosal epithelium (E-cadherin) and the muscular layer (MYH11). White arrows indicate KIT^+^ interstitial cells of Cajal (ICC). Scale bars: 100 μm. Please click here to view a larger version of this figure.

Using the new method, murine gastric Swiss rolls included all three major regions of the stomach: fundus, corpus, and antrum, along with a small portion of the esophagus and the duodenum in a single section. Including the entire stomach in one section allows comprehensive regional analysis (Figure 1). Hematoxylin and eosin (H&E) staining of whole images of the stomach Swiss rolls demonstrated well-preserved gastric architecture across all regions, allowing clear visualization of the mucosal, submucosal, and muscularis layers (Figure 3). Figure 4 shows representative immunofluorescent staining of interstitial cells of Cajal (ICC; pacemaker cells that generate rhythmic slow waves underlying phasic gut motility; identified by KIT and ANO1), enteric neurons (neurons of the enteric nervous system that coordinate gastrointestinal motility; identified by TUBB3 immunostaining), and nuclei (DAPI). In the gastric mucosa, KIT is also expressed in Paneth and goblet cells as previously reported^9,10,11^. ANO1 is also highly expressed in the gastric epithelium, particularly in the columnar epithelial cells of the fundus, consistent with its role in chloride transport and mucosal secretion^11^. In addition, TUBB3 is expressed in certain gastric endocrine cells and mucus-secreting cells, according to data from the Human Protein Atlas^12,13^. Figure 5 shows representative immunofluorescent staining of smooth muscle cells labeled by MYH11, gastric mucosal epithelial cells labeled by E-cadherin, and ICC labeled by KIT. MYH11 is also expressed in myofibroblasts in the gastric mucosa, as reported previously^14^. Together, these immunofluorescence results confirm that our protocol preserves tissue architecture.

## Discussion

Using the newly optimized gastric Swiss-roll method, all three segments of the stomach (fundus, corpus, and antrum), along with the esophagus and duodenum, are included on a single slide. This comprehensive embedding allows researchers to analyze structural and molecular changes throughout the entire stomach and reduces the cost of sectioning and staining reagents (Figure 1).

Exposing all gastric regions to the same staining solutions simultaneously during immunostaining ensures consistency and improves accuracy. Immunostaining of gastric Swiss rolls clearly reveals the distinct layers of the stomach wall. As shown in Figure 4 and Figure 5, this method enables visualization of morphological differences among gastric segments and allows detection of ICC (KIT and ANO1)^10^, neurons (TUBB3)^15^, smooth muscle cells (MYH11), mucosal epithelial cells (E-cadherin), and nuclei.

Minor modifications may be made depending on tissue condition and research purpose. For example, removing extra attached fat can help maintain the roll’s shape during fixation. Adjusting section thickness may also help preserve tissue structure or improve adhesion to the slide. Another potential refinement involves using a metal mold for more consistent cutting, as is done with intestinal Swiss rolls^16^. Although this protocol focuses on cryosections, it may be adapted for paraffin-embedded tissues. While paraffin embedding preserves tissue structural integrity, cryosectioning allows faster processing and better antigen preservation, making it more suitable for immunofluorescence.

This method does have limitations. An enlarged, fibrotic, or calcified stomach, commonly seen in conditions such as inflammation, obesity, diabetes, or aging, can be more difficult to roll and may pose challenges for optimal staining^17,18,19,20^. Despite this limitation, this technique offers major advantages over traditional region-specific embedding. Processing all gastric regions under identical conditions improves consistency, reduces variability, and conserves both time and reagents.

This approach is particularly useful for studies of region-specific change. It also supports spatial analysis of multiple cell types and can be paired with image-based quantification. Overall, this newly optimized gastric Swiss-roll method provides a practical and scalable platform for comprehensive gastric tissue analysis in health and disease.

## Figures and Tables

**Table of Materials:** 

Name of Material/ Equipment	Company	Catalog Number	Comments/Description
Needle 30 G x 1/2 in.	BD Bioscience	305106	
Bovine serum albumin	SIGMA-ALDRICH	A7906	
ColdVision MC-LS LED light source	SCHOTT	MC-LS	
Confocal software	Nikon	Nikon NIS-Elements Imaging Software	
Cryoseal XYL	Epredida	9312-4	
DAPI (4’,6-diamidino-2-phenylindole, dilactate)	Thermo Fisher Scientific	D3571	
Dissection Dishes, Large	Living Systems Instrumentation	SKU: DD-90	
Donkey anti-goat AF647 pAb	Life Technologies	A21447	Lot: 1739289 RRID:AB_2535864
Donkey anti-mouse AF555 pAb	Life Technologies	A31570	Lot: 1774719 RRID:AB_2536180
Donkey anti-rabbit AF488 pAb	Life Technologies	A21206	Lot: 2668665 RRID:AB_2535792
#5SF Forceps	FINE SCIENCE TOOLS	11252-00	
Epoxy Coated Forceps	FINE SCIENCE TOOLS	11210-10	
E-cadharin (1:400 dilution)	BD Bioscience	610181	lot: 25510 RRID:AB_397580
Eosin	ELITechGroup	$SS-071C	
Ethanol	SIGMA-ALDRICH	459828-4L	
Gauze Sponges	Fisherscientific	22-362-178	
FLEX TUBE 1.5 mL	Eppendorf	22364111	
Hardened Fine Scissors	FINE SCIENCE TOOLS	14090-09	
Hematoxylin	Epredia	7211	
KIT (0.2 μg/mL; 1:1000 dilution)	R&D Systems	AF1356	Lot: IEO0217101 RRID: AB_354750
LENS CLEANING SOLUTION 1OZ SPRAY BOTTLE	Nikon	77013066	
Microscope Cover Glass	Fisher Scientific	12541016	
Microscope Slides	CardinalHealth	M6133A	
Minutien Pins	FINE SCIENCE TOOLS	26002-20	
MYH11 (1:400 dilution)	Biomedical Technologies	BT-562	RRID:AB_10013421
Nail polish	SINFUL COLORS	7.33855E+11	
Paraformaldehyde	SIGMA-ALDRICH	158127-500G	
Personna Single Edge Blades	The Razor Blade Co.	76247-652	
ReadyProbes Mouse-on-Mouse IgG Blocking Solution (30X)	Thermo Fisher Scientific	R37621	Lot: 3226230
SlowFade Diamond Antifade Mountant (Mounting media)	Thermo Fisher Scientific	S36972	
StainTray 10 slides staining system	Simport Scientific	M918-2	
Sucrose	SIGMA-ALDRICH	S9378-1KG	
Surface-Amps X-100 (Triton X)	Thermo Fisher Scientific	28314	
Stereo microscope 0.8x–5x	Nikon	SMZ645	
TMEM16A (ANO1: 1:400 dilution)	Abcam	ab53212	Lot: GP3295656-1 RRID: AB_883075
TUBB3 (1:400 dilution)	Cell signaling technology	4466	Lot: 5 RRID: AB_1904176
Transfer Pipet	Falcon	357575	
Xylenes	Fisher Scientific	X3P-1GAL	LOT 206238
